# Potential therapy strategy: targeting mitochondrial dysfunction in sepsis

**DOI:** 10.1186/s40779-018-0187-0

**Published:** 2018-11-26

**Authors:** Hui Zhang, Yong-wen Feng, Yong-ming Yao

**Affiliations:** 1 Trauma Research Center, First Hospital Affiliated to the Chinese PLA General Hospital, Fucheng Road 51, Haidian District, Beijing, 100048 China; 2 Department of Critical Care Medicine, The Second People’s Hospital of Shenzhen, Shenzhen, 518035 China

**Keywords:** Sepsis, Mitochondria, Electron transfer chain, Monitor, Therapy strategy

## Abstract

Recently, the definition of sepsis was concluded to be a life-threatening organ dysfunction caused by a dysregulated host response to infection. Severe patients always present with uncorrectable hypotension or hyperlactacidemia, which is defined as septic shock. The new definition emphasizes dysregulation of the host response and multiple organ dysfunction, which is partially attributed to metabolic disorders induced by energy crisis and oxidative stress. Mitochondria are a cellular organelle that are well known as the center of energy production, and mitochondrial damage or dysfunction is commonly induced in septic settings and is a predominant factor leading to a worse prognosis. In the present review, we determine the major mitochondrial disorders from morphology to functions in sepsis. In the following, several clinical or pre-clinical assays for monitoring mitochondrial function are demonstrated according to accumulated evidence, which is the first step of specific therapy targeting to modulate mitochondrial function. Accordingly, various reagents used for regulating mitochondrial enzyme activities and promoting biogenesis have been documented, among which mitochondria-targeted cation, TPP-conjugated antioxidants are the most valuable for future trials and clinical treatment to improve mitochondrial function as they may take advantage of the prognosis associated with septic complications.

## Background

Sepsis is redefined as life-threatening organ dysfunction caused by a dysregulated host response to infection. Severe patients with septic shock require vasopressors to maintain a mean arterial pressure of 65 mmHg in the absence of hypovolemia or present with hyperlactacidemia (serum lactate level > 2 mmol/L) [1]. A higher serum lactate level reflects a systemic metabolic dysfunction induced by an insufficient consumption of nutrients, such as glucose. Mitochondria are the key cellular organelles responsible for nutrient metabolism and energy production. Sepsis-induced mitochondrial damage or dysfunction is the major cause of cellular metabolism disturbance, insufficient energy production, and accompanied oxidative stress, which evoke apoptosis in both organ cells and immune cells and finally lead to immunologic dissonance, multiple organ failure, and even death in patients [2, 3]. Accordingly, well protection from mitochondrial disorders is critical to reserve cell homeostasis and might be a significant cause of better prognoses.

## Morphology and function of mitochondria

### Morphology

The mitochondrion is a double-membrane-bound organelle found universally in almost all eukaryotic organisms that are commonly between 0.75 and 3.00 μm in diameter but vary in size and structure. The number of mitochondria in a cell may vary widely by cell, tissue or organ type. For instance, red blood cells lack mitochondria, whereas liver cells and skeletal muscle cells can have more than 2000. A mitochondrion is composed of compartments or regions that carry out specialized functions, including the outer membrane, the intermembrane space, the inner membrane, the cristae, and matrix. One of the characteristics of mitochondria that differs from other organelles is that it has an independent genome that shows substantial similarity to bacterial genomes, known as mitochondrial DNA (mtDNA). Mitochondrial proteins transcribed from mtDNA are responsible for its own biogenesis and nutrient metabolism.

### Mitochondrial function

**Fig. 1:**
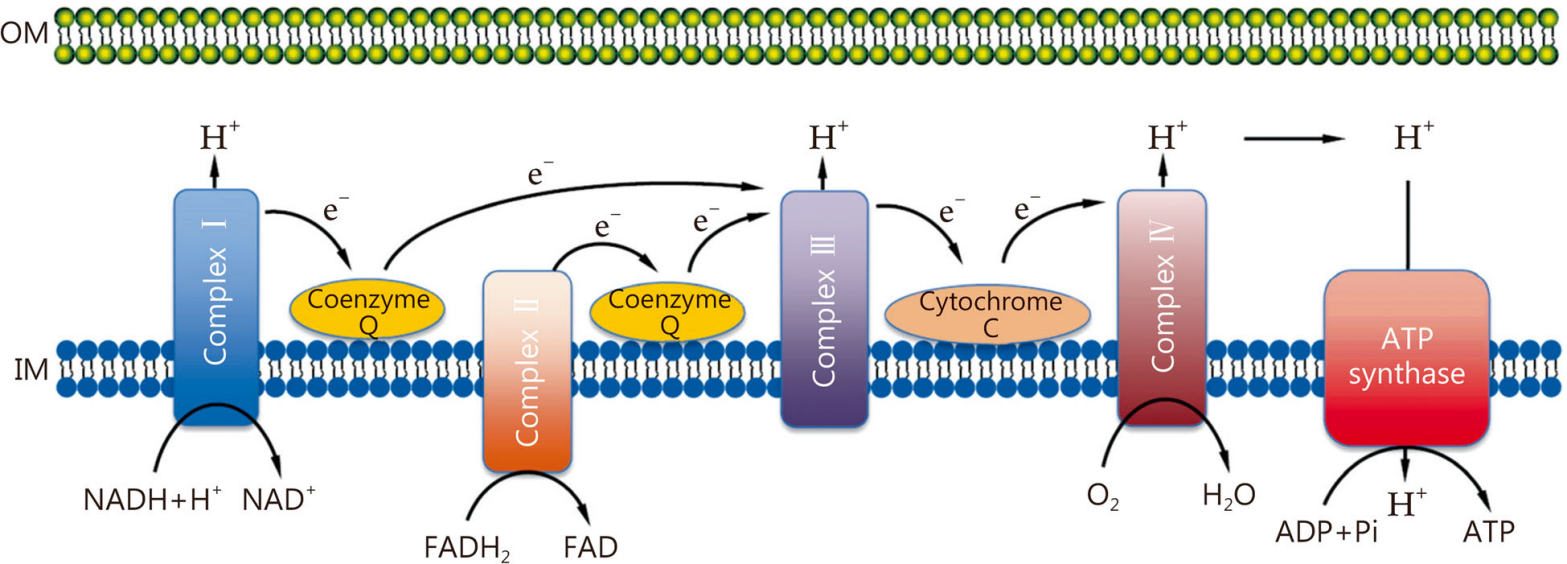
Electron transport chain (ETC) components and its function. NADH and FADH_2_ are produced from the intermediary metabolism of glucose (carbohydrate), lipid (fat), and glutamine (protein); and they donate electrons to complex I (NADH-ubiquinone oxidoreductase) and complex II (succinate-ubiquinone oxidoreductase). These electrons are passed sequentially to coenzyme Q (or ubiquinone) to form CoQH_2_, and then transfers its electron to complex III (ubiquinol-cytochrome C oxidase reductase). Complex III transfers electrons to cytochrome C, which pass them to complex IV (cytochrome C oxidase or COX). At last complex IV donates an electron to O_2_ to produce H_2_O. The energy liberated by the flow of electrons is used by complexes I, III, and IV to pump protons (H^+^) out of the mitochondrial inner membrane (IM) into the intermembrane space. This proton gradient generates the mitochondrial membrane potential that is coupled to ATP (Adenosin triiphosphate) synthesis by complex V (ATPase) from ADP (adenosin diphosphate) and inorganic phosphate (Pi). OM. Outer membrane; NADH. Oxidized nicotinamide adenine dinucleotide; NAD^+^. Reduced nicotinamide adenine dinucleotide; FADH. Oxidized flavin adenine dinucleotide; FAD^+^. Reduced flavin adenine dinucleotide

The dominant roles of mitochondria are to produce the energy currency of the cell, which is also known as ATP through respiration and to regulate cellular metabolism. The central reaction involved in ATP production is the citric acid cycle, which is performed by oxidizing the major products of glucose in the mitochondria matrix. Glucose enters the cellular milieu through glucose transporter 1 (Glut-1), followed by conversion to pyruvate, which is mediated by a series of enzymatic steps, including glucose phosphorylation to glucose-6-phosphate (G-6-P), followed by conversion to pyruvate, reducing NAD^+^ to NADH and generating ATP molecules via oxidative phosphorylation (OXPHOS) through the mitochondrial electron transport chain (ETC). ETC is composed of complex (I, II, III, and IV), coenzyme Q, and cytochrome C, which are located on the mitochondrial inner membrane in sequence and appear to be essential for the generation of mitochondrial membrane potential as well as the proton gradient that is further utilized for the production of ATP at complex V (ATP synthase) (Fig. 1). In addition to the breakdown of glucose via glycolysis, cells have the ability to metabolize other substrates, such as lipids and glutamine, which feed into the citric acid cycle and drive OXPHOS. Fatty acid β-oxidation and glutaminolysis replenish the citric acid cycle intermediates acetyl-CoA and α-ketoglutarate, respectively, thereby fueling oxidative phosphorylation.

Along with bioenergetics, mitochondria are involved in various crucial functions, including redox signaling, calcium flux, and programmed cell death (apoptosis). Mitochondria are the most capable storage for calcium and regulate its intercellular balance. OXPHOS produces reactive oxygen species (ROS) that are indispensable mediators of several signaling pathways. A morphological alteration-induced membrane destabilization or rupture is the promoter for cell apoptosis and calcium disorder, while the dysfunction of ETC-induced incompletion of OXPHOS can cause a lack of energy and overload of ROS, thereby resulting in injury to cells and multiple organs [4, 5].

## Mitochondria dysfunction in sepsis

### Morphological alterations

The description of morphological impairment of mitochondria was first reported in a canine model of septic cardiomyopathy decades ago, and it is characterized by swelling, loss of cristae, cleared matrix, internal vesicles, and rupture of the inner and outer membranes [6]. Similar alterations of mitochondria are identified in septic patients by postmortem biopsies.

The main causes of mitochondrial swelling are collectively known as direct inner membrane damage induced by oxidative stress and calcium overload in the mitochondrial matrix, forcing an increase in membrane pore permeability and a consequent alteration of osmotic pressure between the outer and inner membranes [7, 8]. After injury, dysfunctional or damaged mitochondria are selectively targeted by autophagosomes and delivered to lysosomes for clearance or recycling, which is called mitophagy. In postmortem examination or biopsy in clinical studies, a greater number of autophagosomes are observed in various organ cells in septic patients. A consistent phenomenon has been confirmed in animal experiments, and blockade of autophagy promoted further cell apoptosis and induced target organ damage [9–11]. In contrast, activation of autophagy by rapamycin shows a protective effect on renal function in septic mice [12]. According to the evidence, mitophagy protects cells from apoptosis by clearance of injured mitochondria, which is the main source of ROS and oxidative stress.

Mitochondrial homeostasis requires a perfect equilibrium between mitophagy and mitochondrial biogenesis that is viral for sepsis recovery [13]. Mitochondrial biogenesis is therefore defined as the process by which cells increase their individual mitochondrial mass [14]. However, newly generated mitochondria accumulate in the cytoplasm that are present in various shapes and sizes. Mitochondrial proteins are encoded by either nuclear DNA (nDNA) or mtDNA, which are involved in biogenesis and metabolism. It has been well documented that mtDNA expression is strongly regulated by AMP-activated protein kinase (AMPK), PRARγ-coactivator-1α (PGC-1α), nuclear respiratory factors 1 and 2 (NRF-1and − 2), and mitochondrial transcription factor A (TFAM) [13, 15–17]. A recent report showed a time-dependent activation and nuclear translocation of AMPK and PGC-1α after sepsis in young but not aged mice with significant defect in mitochondrial function. Pharmacological activation of AMPK by AICAR in aged mice protects from liver and cardiac injury, which is associated with improved mitochondrial structure and function [18, 19]. In the early phase of sepsis, mitochondrial biogenesis is activated in mouse kidneys accompanied by active mtDNA expression [20]. Similar results have been reported in skeletal muscle biopsies from patients with sepsis or MODS that showed a marked elevation of mitochondria in the early phase, suggesting activated biogenesis. In addition, the retrospective analysis indicated an elevated expression of PGC-1α in survival patients along with a higher ATP level in muscle cells in comparison with those with fatal outcomes [14].

In the setting of sepsis, both mitophagy and biogenesis are activated to reserve mitochondrial dynamic homeostasis [21, 22]. As a result, increases in mitophgosomes and mitochondria mass with various shapes are observed in cells. This is an essential step in reestablishing energy production and metabolism in cells and organs during recovery from septic response [23–25].

### Disturbance in ETC function

In addition to the morphological change, the function of mitochondria is altered in the development of sepsis, which is mainly due to disturbance of ETC function. During sepsis, inflammatory mediators such as nitric oxide (NO), carbon monoxide, and reactive oxygen/nitrogen species (ROS/NOS) directly impair various components of the mitochondrial ETC complexes and mitochondrial respiration [26–28]. Additionally, a lower metabolic rate in sepsis has been reported and is associated with decreased amounts of mtDNA that regulate the expression of ETC complex components [13].

Clinical data from septic patients show that the extent of mitochondrial impairment in the lungs was correlated with mortality [29]. It documents a dramatic decrement in ETC complex expression, including complex I to IV, and insufficient ATP production in cells of septic patients. Patients who die from severe sepsis show decreased muscle ATP content, while higher levels of ATP are observed in survivors [30]. In a clinical trial, the blood ATP level from critically ill patients was significantly lower than that of healthy volunteers, and it is considered useful as the APACHE II score in evaluating prognosis and morality [31]. Consistent with septic animal models, cardiomyocytes perform much lower ETC complex activities and oxygen consumption. Interestingly, exogenous supplementation of cytochrome c, the coenzyme of complex IV, is effective in improving cardiac function. It is likely that injection of caffeine, which is reported to benefit complex activity, could reserve cardiac systolic function and improve survival [32, 33].

It has been demonstrated that ETC complex activities are suppressed by accumulated ROS in the mitochondria matrix. In septic status, significant elevations of ROS and active nitrogen, including NO, were evident, to which ETC complexes I and IV are extremely sensitive. In an in vitro experiment, Boulos et al. [29] exposed normal vesicular endothelium cells to serum isolated from septic patients; afterwards, ETC complex activities were largely inhibited. However, elimination of NO maintains their activities and ETC function to average range.

**Fig. 2:**
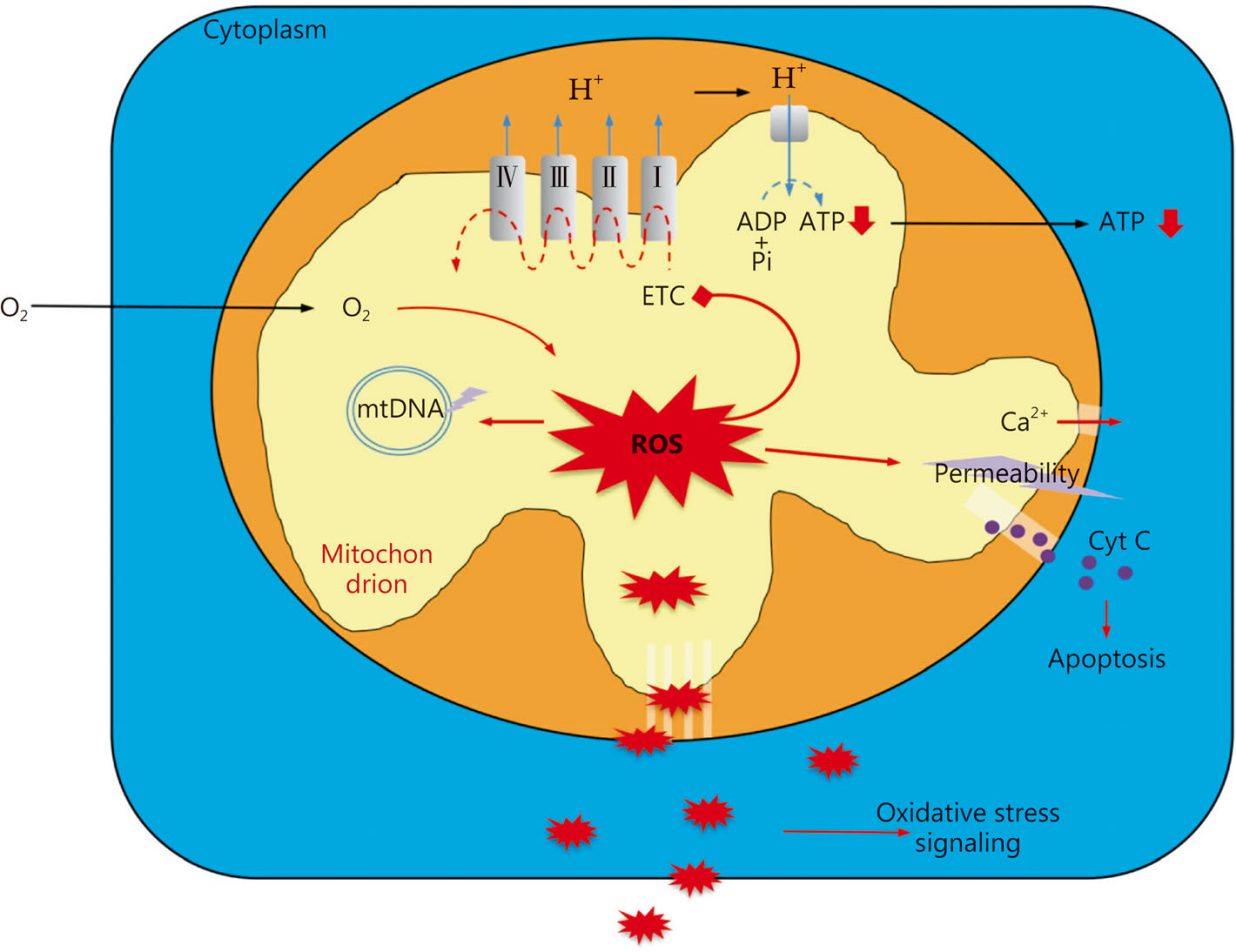
Mitochondria dysfunction in sepsis. The electron transport chain (ETC) dysfunction results in extreme ROS production within mitochondria, which can lead to oxidative damage to mitochondria membrane, ETC activity and mtDNA. Mitochondrial membrane permeability transition results in release of cytochrome C (cyt C) into the cytosol, leading to apoptosis. Increased membrane permeability also makes the Ca^2+^ reflux into cytoplasm and a consequent disturbance that might further activate related signaling pathways. Mitochondrial ROS can also transport to cytoplasm and induce oxidative stress, following by oxidative stress signaling pathways activation which modulate various cellular functions. ROS released into extracellular space will further take harm to other cells and organs

Dysfunction of ETC results in limited ATP production and in the meanwhile produces overload ROS because of the impaired OXPHOS process. ROS accumulation in mitochondria might harm ETC function and membrane permeability, which induce Ca^2+^ reflux and cytochrome c release together with subsequent apoptosis signaling [34, 35]. Moreover, ROS released into the cytoplasm or even the extracellular space is prone to oxidative stress and might consequently induce severe organ injury (Fig. 2).

In addition to OXPHOS in mitochondria, intracellular nutrient metabolism progress has been altered, including glycolysis, fatty acid oxidation and glutaminolysis, which fuel OXPHOS. A recent study reported significant differences in plasma metabolites between sepsis survivors and non-survivors. For example, nine proteins involved in fatty acid transport are decreased in non-survivors, suggesting a defect in fatty acid oxidation. Increased levels of lactate and gluconeogenic amino acids are observed in sepsis non-survivors [36]. These data suggest alterations in nutrient metabolism that might not provide sufficient metabolites to OXPHOS.

### Oxidative stress

Under normal conditions, superoxide (O_2_^−^) is a by-product of ETC in the OXPHOS reaction, transforms to H_2_O_2_ and finally generates H_2_O in the assist with hyperoxydase, such as manganese superoxide dismutase (MnSOD) [37, 38]. In the setting of sepsis, however, ineffective ETC activity is limited to producing ATP but excessive ROS. Consequently, oxidative stress arises as a result of an imbalance between free radical production and antioxidant defense. Accumulated ROS can cause oxidation damage to all cellular components, including lipids, proteins, and DNA [39]. The latter is the most detrimental because replication of damaged DNA can lead to genetic mutations or apoptosis [40]. In addition, extracellular ROS released from dead cells act as an inflammatory mediator causing injury to other cells or organs.

As reported in septic mice, the activity of MnSOD is repressed along with overload hyper oxide accumulation in mitochondria. Treatments with antioxidants reveal significant protective effects on multiple organ failures in septic animals [41]. Additionally, oxidative stress in patients with sepsis has been widely described over recent decades, and the majority of ROS and NOS were generated by damaged mitochondria [42–44]. It is now accepted that oxidative stress plays a central role in the etiology of cell and organ dysfunction and even mortality in sepsis [45–48]. There is a suggestion that progressive improvement in mitochondrial respiration with lower ROS production might be associated with better recovery in organ function in patients who survive sepsis [49].

Collectively, the major morphological and functional alterations of mitochondria are reviewed as above, which are evoked by sepsis and play as critical mediators leading to a worse prognosis. Therefore, early evaluation of mitochondrial function and effective modulation are critical to breaking through the vicious cycle. The therapeutic strategy targeting mitochondrial dysfunction has the potential to improve the prognosis of sepsis.

### Mechanism underlying mitochondria impairment in sepsis

Mitochondria dysfunction during sepsis as described above has been recognized for a long time; however, the underlying mechanism is complicated and waiting for further illustration.

The initial cause might be attributed to hypoxia, which was first identified in the 1940s [50–52]. During sepsis, lower perfusion induced hypoxia augments free radical production because of limited oxygen and incomplete OXPHOS. On the other hand, molecules in the antioxidant system are impaired in both activities and expressions.

The inflammatory cytokines released by activated leukocytes following exposure to DAPMs or PAMPs include lipopolysaccharide (LPS). It has been reported that LPS stimuli induce NAPDH oxidase expression [53]. The cytokines also lead to overproduction of RNS and NO by the promotion of iNOS activity [54–56]. NO can combine with the ROS species peroxide to form the RNS species, which results in irreversible inhibition of ETC activity [57–59]. For example, peroxynitride regulates ETC complex I, resulting in respiratory inhibition and cellular energy decrement, which leads to loss of cell function, as is observed in the heart and skeletal muscle cells in a rodent model of sepsis [60]. Blockade of NO formation by iNOS inhibitors, such as melatonin, has been shown to improve sepsis outcome in both animal models and patients [61–63]. However, recent studies have demonstrated that serum melatonin levels are positively associated with oxidative stress, IL-6 level, SOFA score and mortality in severe septic patients, and non-survivors showed higher serum melatonin [64, 65]. This contradiction may be associated with a decreased utilization of melatonin in mitochondria as an antioxidant.

As a consequence of ETC dysfunction, mitochondria itself becomes a source of excessive ROS generation in sepsis, which in turn inflicts further harm to mitochondria, including injury of the inner membrane, inhibition of ETC activity, and damage to mtDNA. Finally, mitochondria undergo matrix swelling, membrane rupture and initiate apoptosis. Hotchkiss and colleagues first observed high rates of apoptosis in splenic lymphocytes and other organs after sepsis, and inhibition of apoptosis with caspase inhibitors improves survival in sepsis [66, 67]. To adapt cells to wide mitochondrial injury, selective autophagy, mitophagy occurs in the absence of cell apoptosis and is associated with impaired mitochondrial oxygen consumption during sepsis [68]. Meanwhile, mitochondrial biogenesis is activated to compensate for the dramatic loss. LPS has been shown to increase the expression of nuclear respiratory factor-1 (NRF-1) in hepatocytes during sepsis. NRF-1 is a transcriptional activator of TFAM resulting in mtDNA replication and mitochondrial protein synthesis [69]. Evidence has shown a sustained reduction in mitochondrial density after the onset of severe sepsis [70].

In such situations, mitochondrial dysfunction is typically presented as mentioned above. It is worth noting that mitochondrial dysfunction in sepsis is neither a cause nor a consequence; however, it acts as an amplifier in the vicious cycle of sepsis pathophysiology progress.

## Monitor of mitochondrial function

### Potential clinical examinations

Mitochondria are a sub-cellular organelle, and their function is difficult to detect in vivo or implicate in clinical settings. The most widely used assay is spectrometry evaluation of ETC enzyme activities. However, further clinical implication is dependent on the sampling methods. As reported, NADH and ETC complex I~IV activities in platelets were much lower in septic patients [71]. Because of the convenience in peripheral blood sampling, it is available to be implicated in clinical practice. However, the amounts of platelets in septic patients are reduced due to the excessive assumption of disturbed coagulation. Regretfully, after normalized analysis with platelet amount, these index activities do not show significant correlations with the outcome [72].

Another reported method using peripheral blood samples is high-resolution respirometer, which measures the respiratory rate of platelets [73]. The results indicate a gradual decrease in respiratory rate along with aggravation of sepsis and an extreme decline in dead patients. However, the result does not show a significant correlation with the SOFA score. The common and convenient advantage of the above methods is blood sampling, which determines the feasibility of clinical use. The latter detection reserves intact platelets and performs in the patient’s own serum, which mimics the in vivo micro-circumstance and reflects cellular respiratory function much more preciously. However, according to the presented reports, neither is confirmed to evaluate the development or prognosis of sepsis. Further sufficient and reliable clinical data are required for these potent implications.

### Pre-clinical applications

In addition, several in vivo detection methods of mitochondrial function have already been implicated in animal models.

ATP production is the final event of OXPHOS and is a suitable biomarker for intact mitochondrial function. The P^32^-labeled ATP assay by magnetic resonance spectrum (MRS) has been used in animal models to determine ATP generation in vivo [74, 75]. However, in a clinical study, the ATP content in immune cells from peripheral blood sampling did not reveal differences compared to healthy volunteers [76, 77]. For further convenient clinical use, specific chemosensors of ATP have been developed as fluorescence probes, which are capable of measuring ATP locating on polymorphonuclear neutrophil (PMN) membranes and in mitochondria matrix, respectively (PMAP-1 and MitoAP-1). Contrary to a previous study, the levels of MitoAP-1 in PMNs from septic patients were higher than in healthy controls, suggesting an elevation of ATP production in mitochondria. The study included very few patients observing 3–4 days after in-hospital; thus, the limited data might not present the dynamic change in ATP production and mitochondrial function in aggravation of sepsis. Nevertheless, the mitochondrial ATP assay method is available for further implication in clinical settings [78].

Other indirect markers used in animal models are designed to target mitochondrial enzymes, such as NAPDH. Its autofluorescence absorption is measured at a wavelength of 450 nm in comparison to NAD^−^ at 340 nm. Evaluation of the NADH/NAD^−^ ratio according to the difference reflects the activity of ETC [79–82]. Infrared ray analysis is also implicated in evaluating ETC function. Cu A, the core of cyclooxygenase, can be absorbed at 830 nm only after oxidation, which is a likely indicator of ETC function and cellular oxygen assumption [83–86].

Although these methods can be performed easily and quickly in animal models, their safety and validity for clinical implications require further development and verification.

## Potential mitochondria-targeted therapeutic strategies

### Mitochondria membrane stabilization

Mitochondria membrane permeability increases under oxidative stress or other inducers, which accounts for the persistent opening of multiple channels, including voltage-dependent anion channels (VDACs) located on the outer membrane and K-ATP channels on the inner membrane. Apoptotic signals promote VADC opening through the up-regulation of Bax expression and translocation on the outer membrane, in turn leading to cytochrome C leakage to the cytoplasm and activating canonical apoptosis via caspase cleavage [87, 88]. Accordingly, blockade or interference with the pre-mitochondrial apoptotic pathway can protect against outer membrane breakdown. For the regulation of inner membrane K-ATP channels, an effective blocker, 5-hydroxydecanoate (5-HD), has been reported to protect mitochondria permeability after inner membrane injury, prevent ATP reflux and further mitochondrial swelling and rupture. In septic rats, severe mitochondrial rupture is observed in cardiomyocytes, accompanied by increased cytochrome C in the cytoplasm. Treatment with 5-HD preserves membrane permeability and integrity, which also drastically reduces animal mortality [89].

In contrast, levosimendan, a calcium sensitizer, which was used as a vasodilator by opening the K-ATP channel, has been reported as an effective drug for sepsis. Some clinical trials show consistent reductions in sepsis patient mortality [90]. A recent clinical trial indicated that levosimendan might improve cellular metabolic alterations in patients with septic shock [91]. The effect of levosimendan is not specific to regulating mitochondrial channels, and the protective role might also be due to its antioxidant action by increasing antioxidant defense and other unrelated mitochondrial effects [92]. Regardless, levosimendan is a potential reagent for sepsis treatment.

### Reservation of ETC function

ETC dysfunction is the major cause of insufficient energy production but adequate ROS in mitochondria. To solve these problems, exogenous ATP was supplied to septic animals, while it did not provide satisfactory improvement. ROS overload and the subsequent oxidative stress are key factors that lead to further mitochondrial injury and severe damage in whole target organs working as a vicious cycle. In the early decades, enzyme supplementation has already been used to improve ETC function, such as coenzyme Q (CoQ10, also called Quinone). A clinical trial has shown that exogenous administration of ubiquinol (the reduced form of CoQ10) could increase plasma CoQ10 levels in septic patients, but the outcomes were not different from the placebo group [93]. These reagents are rarely concentrated in the mitochondria matrix or the intermembrane space where they can work to improve ETC function. Therefore, treatment with such traditional reagents is not satisfactory.

Antioxidant molecules can be covalently attached to lipophilic cations, which accumulate in mitochondria as a result of the mitochondrial membrane potential. In recent years, triphenylphosphonium (TPP) has been discovered. The negative charge inside the mitochondrial inner membrane results in the TPP conjugated antioxidants accumulating within mitochondria to approximately 500 times the levels in the cytoplasm [94]. Such types of reagents under well development include MitoQ (TPP covalent Quinone) and MitoE (Vitamin E). Their concentrations in mitochondria are dramatically elevated compared with traditional enzyme supplementation, and TPP conjugation does not affect enzyme activities.

Once inside the mitochondria, the MitoQ is translocated onto the inner membrane and is recycled to active ubiquinol in the respiratory chain. In septic animal models, MitoQ intakes obviously improved ETC function, showing a large increase in complex I–IV activities [95]. Mitochondrial targeting quinone supplementation can assist electron transportation through ETC with sufficient ATP output and is also helpful to reduce ROS production, which might block the vicious cycle of mitochondria injury-induced cell damage. It has been investigated in both in vitro studies and animal models, including hypertension-induced heart failure, lung injury, liver disease diabetes, acute kidney injury, and Alzheimer’s disease.

To date, only two phase II clinical trials using MitoQ have been completed. One is designed to treat Alzheimer’s disease with a continuous oral intake of MitoQ for a whole year, but they show no difference on any measure of progression by clinical scores compared with the placebo controls [96]. The other is used for the treatment of hepatitis virus C infection. After a 28-day intake of MitoQ (40 or 80 mg/d), only plasma alanine aminotransferase levels slightly decreased without a significant difference in HCV load [97]. Unfortunately, the above clinical trials have not achieved satisfaction. Nonetheless, their conclusions take into account the involvement of mitochondrial dysfunction and oxidative stress in the disease. For sepsis, especially during the early phase, mitochondria injury and overload ROS are predominantly harmful factors in mediating the host response to infections. Thus, MitoQ might perform a better effect on the treatment of septic complications.

MitoE, a form of Vitamin E attached to the TPP cation, has been documented in vitro to promote mitochondrial biogenesis, protect mitochondria and whole cells from oxidative stress, and be much more effective than non-targeted equivalents [98–101]. Other compounds have also been conjugated to TPP, e.g., the peroxidase compound Ebselen, called Mito-Peroxidase. In contrast to MitoQ and MitoE, Mito-Peroxidase was only slightly more effective than the non-targeted form in preventing oxidative stress-induced mitochondrial damage because its accumulation in mitochondria seemed to be less pronounced [102]. MitoTEMPO is a TPP-based nitroxide that works as a hydrophilic SOD mimetic specifically in the mitochondrial matrix. Plastoquinone is a plant quinone involved in photosynthesis, which is attached to the TPP cation to form a molecule called SkQ1 as an alternative to MitoQ. MitoTEMPO and SkQ1 also protect cells against oxidative stress both in vitro and in vivo, including in septic models [103–105].

Although limited clinical trial data have not presented satisfactory therapeutic effects, the safety of clinical use is already confirmed, and its implication in septic animals has indicated an exact target organ protective effect. In the coming future, clinical trials in the treatment of sepsis are under expectation.

### Biogenesis promotion

Autophagy is activated after irreversible mitochondrial damage for clearance, while mitochondrial biogenesis is activated through the AMPK/PGC-1a/NRF-1/2 signaling pathway. Insufficient ATP production resulted in ATP/ADP ratio disturbance-activated AMPK and the following PGC-1a/NRF-1/2 pathway, consequently contributing to TFAM expression. TFAM is a promoter of mtDNA expression after its translocation into the mitochondrial matrix and evokes its biogenesis. In both septic patients and animal models, enhanced PGC-1α expression is consistently observed and correlated with a better prognosis. However, AMPK/PGC-1α signaling has a universal effect on cell biology, and its targeting therapeutic strategy might lead to other unbeneficial effects. As a result, much more specific treatment targeted to TFAM is reliable. Currently, recombinant human TFAM (rhTFAM) has been generated and performs well in animal experiments. The impact of rhTFAM has been identified to increase mtDNA expression and improve mitochondrial function in various target organs. Furthermore, it can effectively pass through the blood-brain barrier and protect multiple organs from endotoxin challenge, such as the brain, heart, lung, liver and kidney, accompanied by reduced mortality in septic animals [106].

## Conclusions

**Fig. 3:**
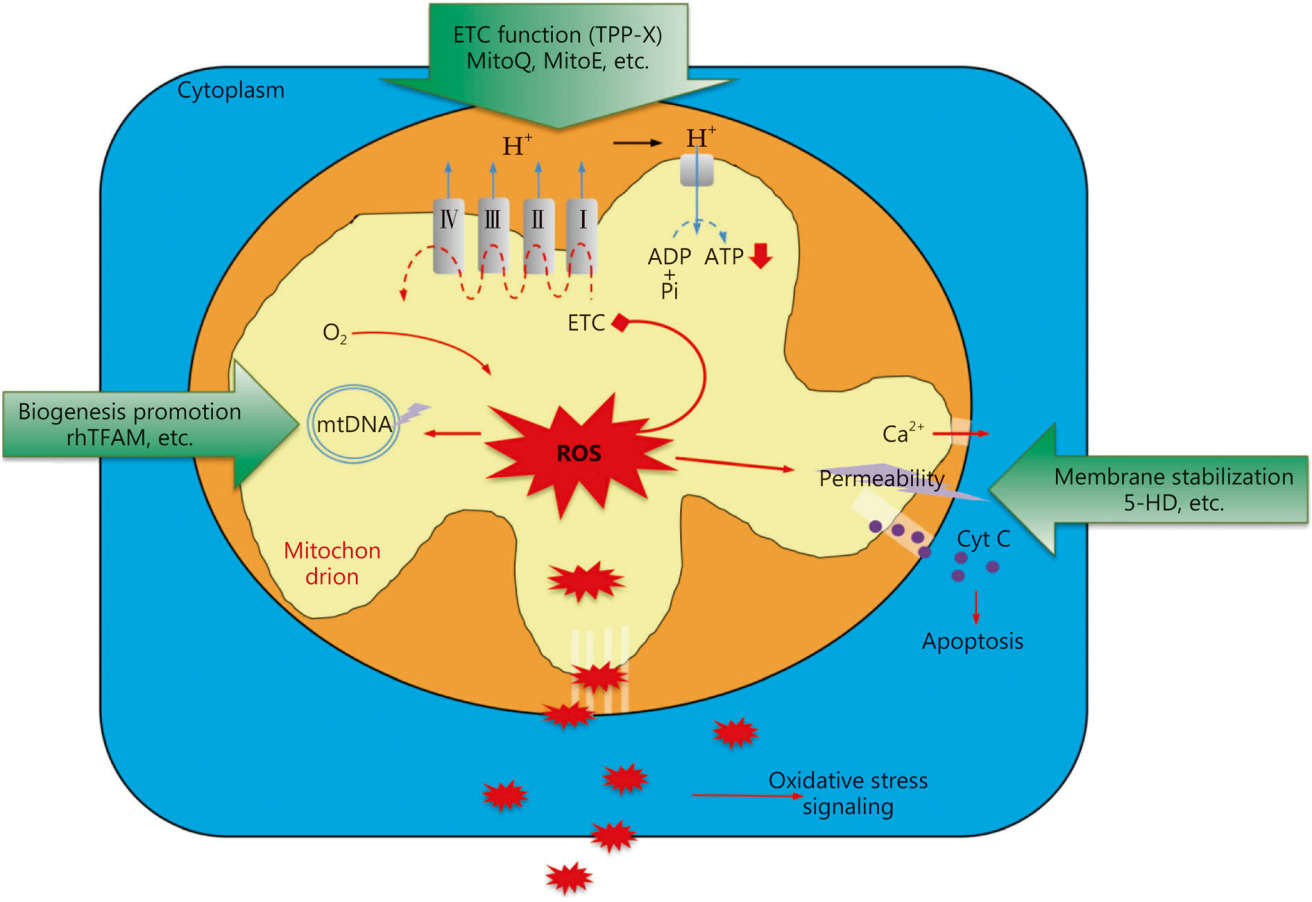
Mitochondria targeted therapeutic strategies. The most potential therapy is TPP cation conjugated antioxidants including MitoQ, MitoE, MitoPrrioxidase, MitoTEMPO, and SKQ1, etc., which specifically accumulate into mitochondria and improve ETC function. Membrane stabilization inhibits ROS induced further membrane injury, protects mitochondria from swelling and rupture, and reduce molecule leakage that causes apoptosis and calcium disturbance in cytoplasm. The reagents include K-ATP channel blocker, 5-hydroxydecanoate (5-HD), etc. Mitochondrial biogenesis promotion by recombinant human mitochondrial transcription factor A (rhTFAM) reactivates mtDNA expressions, thereby enhancing mitochondrial protein expressions

Mitochondrial dysfunction is critically involved in the pathogenesis of sepsis, especially severe sepsis and septic shock and is a predominant factor associated with poor outcome, including multiple organ failure and even death. It is still difficult to illustrate whether mitochondrial dysfunction is a consequence or an inducer in the development of the septic response. Regardless, it is an indispensible factor in a vicious cycle leading to a worse prognosis. Thus, in the future, breakdown of the vicious cycle through modulating mitochondrial function is a potential therapeutic strategy in the management of sepsis (Fig. 3).

However, mitochondrial dysfunction involves many inducible factors, among which specific and effective targets are required for further clinical use. Unspecific therapies such as apoptosis inhibitors, autophagy promoters or hormone treatment might bring universal effects to other cell biological processes and cause unpredictable consequences, which are not suitable for clinical application.

According to the above review, the most potential clinical reagents are TPP cation-conjugated antioxidants, which are targeted to improve mitochondrial enzyme activities. MitoQ, short for TPP conjugated coenzyme Q, is in commercial use to target antioxidation and aging. However, the related clinical trials are limited in treating Alzheimer’s disease and HCV infection and have not acquired satisfactory results in prognosis, although the parameters of mitochondrial enzyme activities are improved to a certain extent. For further trials, especially for the treatment of sepsis, its effective dose and probable administration method still require investigation in a large number of clinical patients.

Theoretically, in addition to improving mitochondrial function, biogenesis activation is another feasible strategy for protecting mitochondrial homeostasis and might benefit the prognosis of sepsis. rhTFAM is already used for animal experiments, and its protective effect on mitochondria was dose-dependent, which might cause extreme biogenesis within high-dose and down-regulate overload ROS as well as autophagy activation. As a consequence, in further pre-clinical and clinical trials, effective and protective dosage is a critical issue waiting for solution.

The major function of mitochondria is energy production relying on the intact OXPHOS system, which involves various molecules and enzymes. In this regard, a combination of multiple specific targeting reagents might achieve better therapeutic effects through multiple aspects to improve mitochondrial function. However, such positive evidence is still lacking and requires further investigation.

In addition to mitochondrial function reservation, nutrition support is indispensable for sepsis recovery because it provides the source of energy production. Taken together, in the base of sufficient nutrition support, healthy mitochondrial function is critical to produce energy and limit ROS generation, both of which are significant for reserving not only mitochondria but also the whole immune system as well as host homeostasis, contributing to the successful modulation of the septic response.

## Abbreviations

5-HD: 5-hydroxydecanoate
AMPK: AMP-activated protein kinase
ETC: Electron transport chain
G-6-P: Glucose-6-phosphate
Glut-1: Glucose transporter 1
LPS: Lipopolysaccharide
MnSOD: Manganese superoxide dismutase
MRS: magnetic resonance spectrum
mtDNA: Mitochondrial DNA
nDNA: Nuclear DNA
NO: nitric oxide
NOS: nitrogen species
NRF-1: Nuclear respiratory factor-1
NRF-1/2: nuclear respiratory factors 1 and 2
OXPHOS: Oxidative phosphorylation
PGC-1α: PRARγ-coactivator-1α
PMN: Polymorphonuclear neutrophil
rhTFAM: Recombinant human TFAM
ROS: Reactive oxygen species
TFAM: Mitochondrial transcription factor A
TPP: Triphenylphosphonium
VDACs: Voltage dependent anion channels
